# 11β,17,21-Trihydr­oxy-6α-methyl-3,20-dioxopregna-1,4-dien-21-yl 3-carboxy­propionate

**DOI:** 10.1107/S1600536809011969

**Published:** 2009-04-08

**Authors:** Hui-Mei An, Ning-Bo Gong, Yang Lu

**Affiliations:** a Institute of Materia Medica, Chinese Academy of Medical Sciences and Peking Union Medical College, 1 Xiannong tan street, Beijing 100050, People’s Republic of China

## Abstract

The mol­ecule of the title compound, C_26_H_34_O_8_, a prednisolone derivative, contains three six-membered rings (*A*, *B* and *C*) and one five-membered ring (*D*). Ring *A* is planar and rings *B* and *C* adopt chair conformations, while ring *D* adopts an envelope conformation with the C atom bonded to the methyl group at the flap. The crystal structure is stabilized by intermolecular O—H⋯O hydrogen bonds

## Related literature

For the preparation, see: Anderson *et al.* (1984 ▶). For bond-length data, see Allen *et al.* (1987 ▶).
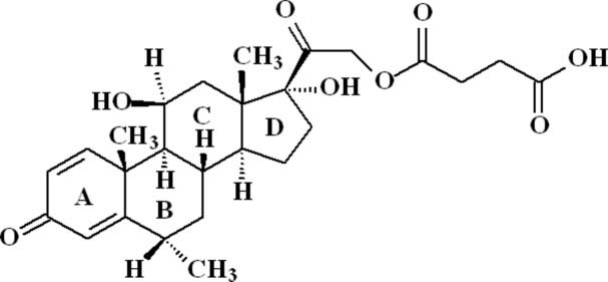


## Experimental

### 

#### Crystal data

C_26_H_34_O_8_
*M* *_r_* = 474.53
Orthorhombic, 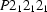

*a* = 8.3125 (1) Å
*b* = 10.1765 (1) Å
*c* = 28.8472 (3) Å
*V* = 2440.25 (5) Å^3^
*Z* = 4
Cu *K*α radiation
μ = 0.79 mm^−1^
*T* = 296 K
0.30 × 0.20 × 0.20 mm

#### Data collection

Bruker SMART APEX diffractometer
Absorption correction: none
8346 measured reflections
3397 independent reflections
3365 reflections with *I* > 2σ(*I*)
*R*_int_ = 0.018
θ_max_ = 58.8°

#### Refinement

*R*[*F* ^2^ > 2σ(*F* ^2^)] = 0.038
*wR*(*F* ^2^) = 0.101
*S* = 1.07
3397 reflections
309 parameters
H-atom parameters constrained
Δρ_max_ = 0.24 e Å^−3^
Δρ_min_ = −0.13 e Å^−3^
Absolute structure: Flack (1983 ▶), 1388 Friedel pairs
Flack parameter: 0.0 (2)

### 

Data collection: *SMART* (Bruker, 2005 ▶); cell refinement: *SAINT* (Bruker, 2005 ▶); data reduction: *SAINT*; program(s) used to solve structure: *SHELXS97* (Sheldrick, 2008 ▶); program(s) used to refine structure: *SHELXL97* (Sheldrick, 2008 ▶); molecular graphics: *SHELXTL* (Sheldrick, 2008 ▶); software used to prepare material for publication: *SHELXL97*.

## Supplementary Material

Crystal structure: contains datablocks I, global. DOI: 10.1107/S1600536809011969/sj2598sup1.cif

Structure factors: contains datablocks I. DOI: 10.1107/S1600536809011969/sj2598Isup2.hkl

Additional supplementary materials:  crystallographic information; 3D view; checkCIF report

## Figures and Tables

**Table 1:** Hydrogen-bond geometry (Å, °)

*D*—H⋯*A*	*D*—H	H⋯*A*	*D*⋯*A*	*D*—H⋯*A*
O2—H2*A*⋯O4^i^	0.82	2.30	3.115 (2)	172
O3—H3*A*⋯O7^ii^	0.82	2.13	2.943 (2)	173
O8—H8*B*⋯O1^iii^	0.82	1.82	2.640 (3)	176

Symmetry codes: (i) 
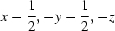
; (ii) 
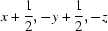
; (iii) 
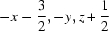
.

**Fig. 1.**
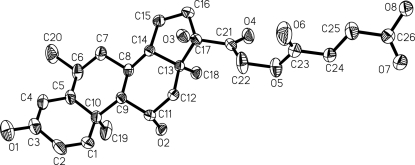
View of the molecule of (I) showing the atom-labelling scheme. Displacement ellipsoids are drawn at the 30% probability level. H atoms have been omitted.

**Fig. 2.**
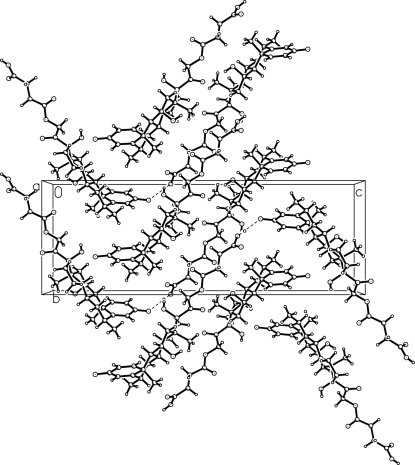
The molecular packing of (I) viewed along the a axis. Dashed lines indicate the hydrogen bonding interactions.

## supplementary crystallographic information

### Comment

The title compound,(I)(Fig. 1),is a prednisolone derivative. It is prepared according to the procedure of Anderson *et al.* (Anderson *et al.*, 1984) and recrystallized from methanol. Its crystal structure is reported here for the first time, Fig, 1.

Bond lengths within the molecule are normal (Allen *et al.*, 1987). The molecule contains three six-membered rings(A/B/C) and one five-membered ring (D). Ring A is planar and ring B and C adopt chair conformations, while ring *D* adopts an envelope conformation wit hatom C13 at the flap. Rings B, *C* and D are *trans*-fused. The dihedral angles between the least-squares planes fitted through all non-H atoms of the rings are A/B = 139.0 (2) °,B/C = 5.9 (2) °, C/D = 7.3 (3) °.

The structure is stablized by intermolecular hydrogen bonds (Table 2), which link the molecules into infinite chains. The hydrogen-bonding arrangement is shown in Fig.2.

### Experimental

The title compound was prepared according to the procedure of Anderson *et al.* (1984). Crystals suitable for data collection were obtained by slow evaporation from methanol solution at 283 K over a period of two weeks.

### Refinement

In the presence of significant anomalous scattering effects, Friedel pairs were not merged. the absolute configuration was established based on the Flack parameter 0.0 (2). All the H atoms were constrained to an ideal geometry with C—H distances of 0.98 Å and *U*_iso_(H) = 1.2*U*_eq_(C)for CH; 0.97 Å and *U*_iso_(H) = 1.2*U*_eq_ (C)for CH_2_; 0.96 Å and *U*_iso_(H) = 1.5*U*_eq_(C)for CH_3_; and 0.82 Å and *U*_iso_(H) = 1.5*U*_eq_(C)for OH atoms.

### Crystal data

**Table:**

C_26_H_34_O_8_	*F*(000) = 1016
*M**_r_* = 474.53	*D*_x_ = 1.292 Mg m^−^^3^
Orthorhombic, *P*2_1_2_1_2_1_	Cu *K*α radiation, λ = 1.54178 Å
Hall symbol: P 2ac 2ab	Cell parameters from 8346 reflections
*a* = 8.3125 (1) Å	θ = 3.1–58.8°
*b* = 10.1765 (1) Å	µ = 0.79 mm^−^^1^
*c* = 28.8472 (3) Å	*T* = 296 K
*V* = 2440.25 (5) Å^3^	Block, colorless
*Z* = 4	0.30 × 0.20 × 0.20 mm

### Data collection

**Table:**

Bruker SMART APEX diffractometer	3365 reflections with *I* > 2σ(*I*)
Radiation source: fine-focus sealed tube	*R*_int_ = 0.018
graphite	θ_max_ = 58.8°, θ_min_ = 3.1°
φ and ω scans	*h* = −9→6
8346 measured reflections	*k* = −11→10
3397 independent reflections	*l* = −32→30

### Refinement

**Table:**

Refinement on *F*^2^	Hydrogen site location: inferred from neighbouring sites
Least-squares matrix: full	H-atom parameters constrained
*R*[*F*^2^ > 2σ(*F*^2^)] = 0.038	*w* = 1/[σ^2^(*F*_o_^2^) + (0.0538*P*)^2^ + 0.6209*P*] where *P* = (*F*_o_^2^ + 2*F*_c_^2^)/3
*wR*(*F*^2^) = 0.101	(Δ/σ)_max_ < 0.001
*S* = 1.07	Δρ_max_ = 0.24 e Å^−^^3^
3397 reflections	Δρ_min_ = −0.13 e Å^−^^3^
309 parameters	Extinction correction: *SHELXL97* (Sheldrick, 2008), Fc^*^=kFc[1+0.001xFc^2^λ^3^/sin(2θ)]^-1/4^
0 restraints	Extinction coefficient: 0.0070 (5)
Primary atom site location: structure-invariant direct methods	Absolute structure: Flack (1983), 1388 Friedel pairs
Secondary atom site location: difference Fourier map	Flack parameter: 0.0 (2)

### Special details

**Table:**

Geometry. All e.s.d.'s (except the e.s.d. in the dihedral angle between two l.s. planes) are estimated using the full covariance matrix. The cell e.s.d.'s are taken into account individually in the estimation of e.s.d.'s in distances, angles and torsion angles; correlations between e.s.d.'s in cell parameters are only used when they are defined by crystal symmetry. An approximate (isotropic) treatment of cell e.s.d.'s is used for estimating e.s.d.'s involving l.s. planes.
Refinement. Refinement of *F*^2^ against ALL reflections. The weighted *R*-factor *wR* and goodness of fit *S* are based on *F*^2^, conventional *R*-factors *R* are based on *F*, with *F* set to zero for negative *F*^2^. The threshold expression of *F*^2^ > σ(*F*^2^) is used only for calculating *R*-factors(gt) *etc*. and is not relevant to the choice of reflections for refinement. *R*-factors based on *F*^2^ are statistically about twice as large as those based on *F*, and *R*- factors based on ALL data will be even larger.

### Fractional atomic coordinates and isotropic or equivalent isotropic displacement parameters (Å^2^)

**Table:**

	*x*	*y*	*z*	*U*_iso_*/*U*_eq_	
O1	−0.8924 (3)	−0.6628 (2)	−0.32497 (7)	0.0955 (7)	
O2	−0.90863 (19)	−0.50797 (16)	−0.09117 (6)	0.0600 (4)	
H2A	−0.9235	−0.4642	−0.0677	0.090*	
O3	−0.5087 (2)	−0.12455 (15)	−0.12292 (5)	0.0553 (4)	
H3A	−0.4421	−0.0651	−0.1224	0.083*	
O4	−0.4761 (2)	−0.1328 (2)	−0.00271 (6)	0.0762 (6)	
O5	−0.7026 (3)	0.04879 (18)	−0.00567 (7)	0.0825 (6)	
O6	−0.5875 (5)	0.2234 (3)	−0.03398 (9)	0.1343 (13)	
O7	−0.7607 (2)	0.41973 (19)	0.11378 (7)	0.0790 (6)	
O8	−0.5211 (2)	0.51291 (17)	0.10461 (6)	0.0658 (5)	
H8B	−0.5516	0.5577	0.1265	0.099*	
C1	−0.9685 (4)	−0.5852 (4)	−0.20758 (11)	0.0896 (10)	
H1A	−1.0508	−0.5488	−0.1899	0.107*	
C2	−0.9911 (5)	−0.5948 (4)	−0.25305 (13)	0.0949 (11)	
H2B	−1.0868	−0.5647	−0.2660	0.114*	
C3	−0.8704 (4)	−0.6509 (3)	−0.28248 (9)	0.0689 (7)	
C4	−0.7268 (4)	−0.6966 (3)	−0.26107 (8)	0.0641 (7)	
H4A	−0.6489	−0.7351	−0.2798	0.077*	
C5	−0.6968 (3)	−0.6878 (2)	−0.21540 (8)	0.0574 (6)	
C6	−0.5400 (4)	−0.7281 (3)	−0.19360 (9)	0.0702 (7)	
H6A	−0.5652	−0.7894	−0.1684	0.084*	
C7	−0.4647 (3)	−0.6065 (2)	−0.17175 (8)	0.0585 (6)	
H7A	−0.4334	−0.5465	−0.1963	0.070*	
H7B	−0.3677	−0.6325	−0.1554	0.070*	
C8	−0.5745 (3)	−0.5339 (2)	−0.13803 (7)	0.0457 (5)	
H8A	−0.5897	−0.5879	−0.1102	0.055*	
C9	−0.7399 (3)	−0.5045 (2)	−0.15972 (7)	0.0449 (5)	
H9A	−0.7170	−0.4452	−0.1856	0.054*	
C10	−0.8194 (3)	−0.6292 (3)	−0.18273 (8)	0.0614 (7)	
C11	−0.8511 (3)	−0.4250 (2)	−0.12729 (7)	0.0480 (5)	
H11A	−0.9444	−0.3969	−0.1456	0.058*	
C12	−0.7685 (3)	−0.3014 (2)	−0.10898 (7)	0.0455 (5)	
H12A	−0.8361	−0.2620	−0.0854	0.055*	
H12B	−0.7583	−0.2387	−0.1341	0.055*	
C13	−0.6016 (2)	−0.3268 (2)	−0.08837 (7)	0.0404 (5)	
C14	−0.5006 (3)	−0.4029 (2)	−0.12406 (7)	0.0419 (5)	
H14A	−0.4994	−0.3489	−0.1522	0.050*	
C15	−0.3304 (3)	−0.3969 (3)	−0.10427 (8)	0.0555 (6)	
H15A	−0.2510	−0.4013	−0.1289	0.067*	
H15B	−0.3119	−0.4687	−0.0828	0.067*	
C16	−0.3219 (3)	−0.2647 (2)	−0.07924 (8)	0.0553 (6)	
H16A	−0.2436	−0.2078	−0.0940	0.066*	
H16B	−0.2909	−0.2771	−0.0471	0.066*	
C17	−0.4916 (3)	−0.2038 (2)	−0.08224 (7)	0.0451 (5)	
C18	−0.6113 (3)	−0.3993 (2)	−0.04181 (7)	0.0522 (5)	
H18A	−0.6789	−0.4752	−0.0450	0.078*	
H18B	−0.6557	−0.3417	−0.0188	0.078*	
H18C	−0.5055	−0.4262	−0.0325	0.078*	
C19	−0.8682 (6)	−0.7359 (3)	−0.14756 (11)	0.1111 (15)	
H19A	−0.9158	−0.8087	−0.1637	0.167*	
H19B	−0.9445	−0.7002	−0.1260	0.167*	
H19C	−0.7746	−0.7654	−0.1311	0.167*	
C20	−0.4209 (5)	−0.7970 (4)	−0.22589 (13)	0.1146 (14)	
H20A	−0.4703	−0.8740	−0.2388	0.172*	
H20B	−0.3269	−0.8219	−0.2087	0.172*	
H20C	−0.3908	−0.7383	−0.2505	0.172*	
C21	−0.5369 (3)	−0.1198 (2)	−0.04012 (8)	0.0492 (5)	
C22	−0.6658 (5)	−0.0208 (3)	−0.04765 (10)	0.0921 (11)	
H22A	−0.6314	0.0412	−0.0712	0.110*	
H22B	−0.7619	−0.0646	−0.0588	0.110*	
C23	−0.6483 (3)	0.1700 (3)	−0.00242 (9)	0.0626 (6)	
C24	−0.6815 (3)	0.2305 (2)	0.04364 (9)	0.0610 (6)	
H24A	−0.7964	0.2448	0.0468	0.073*	
H24B	−0.6484	0.1702	0.0679	0.073*	
C25	−0.5967 (4)	0.3568 (3)	0.04982 (9)	0.0732 (8)	
H25A	−0.6204	0.4122	0.0233	0.088*	
H25B	−0.4819	0.3398	0.0496	0.088*	
C26	−0.6364 (3)	0.4306 (2)	0.09245 (8)	0.0535 (6)	

### Atomic displacement parameters (Å^2^)

**Table:**

	*U*^11^	*U*^22^	*U*^33^	*U*^12^	*U*^13^	*U*^23^
O1	0.141 (2)	0.0889 (15)	0.0562 (11)	−0.0046 (15)	−0.0195 (12)	0.0014 (10)
O2	0.0627 (9)	0.0618 (9)	0.0556 (9)	−0.0188 (8)	0.0214 (8)	−0.0138 (8)
O3	0.0654 (10)	0.0508 (9)	0.0496 (8)	−0.0166 (8)	−0.0041 (7)	0.0053 (7)
O4	0.0738 (11)	0.0993 (15)	0.0554 (10)	0.0139 (11)	−0.0210 (9)	−0.0230 (10)
O5	0.1149 (16)	0.0537 (10)	0.0791 (12)	0.0066 (11)	0.0027 (11)	−0.0234 (9)
O6	0.203 (3)	0.117 (2)	0.0835 (15)	−0.056 (2)	0.0512 (19)	−0.0292 (15)
O7	0.0697 (12)	0.0676 (11)	0.0996 (14)	−0.0008 (10)	0.0157 (11)	−0.0215 (10)
O8	0.0699 (11)	0.0586 (10)	0.0689 (11)	−0.0049 (9)	0.0074 (9)	−0.0096 (8)
C1	0.0519 (15)	0.121 (3)	0.096 (2)	−0.0204 (16)	0.0057 (14)	−0.055 (2)
C2	0.0768 (19)	0.111 (3)	0.097 (2)	−0.0051 (19)	−0.0278 (17)	−0.046 (2)
C3	0.094 (2)	0.0590 (15)	0.0539 (15)	−0.0130 (15)	−0.0066 (14)	−0.0067 (12)
C4	0.0861 (18)	0.0610 (14)	0.0452 (12)	−0.0075 (13)	0.0079 (12)	−0.0080 (11)
C5	0.0817 (17)	0.0459 (12)	0.0446 (12)	−0.0125 (12)	0.0056 (12)	−0.0072 (10)
C6	0.096 (2)	0.0558 (15)	0.0586 (14)	0.0148 (14)	−0.0012 (14)	−0.0162 (12)
C7	0.0661 (15)	0.0590 (15)	0.0504 (12)	0.0117 (12)	−0.0005 (11)	−0.0078 (11)
C8	0.0581 (12)	0.0425 (11)	0.0366 (10)	0.0012 (10)	0.0010 (9)	0.0010 (9)
C9	0.0526 (12)	0.0475 (11)	0.0346 (10)	−0.0101 (10)	0.0026 (9)	0.0010 (9)
C10	0.0707 (16)	0.0671 (15)	0.0463 (12)	−0.0268 (13)	0.0154 (11)	−0.0148 (12)
C11	0.0427 (11)	0.0522 (12)	0.0491 (12)	−0.0067 (10)	0.0006 (9)	−0.0028 (10)
C12	0.0429 (11)	0.0462 (11)	0.0475 (11)	0.0021 (9)	0.0019 (9)	−0.0050 (9)
C13	0.0444 (11)	0.0423 (10)	0.0347 (9)	−0.0016 (9)	0.0021 (9)	−0.0032 (8)
C14	0.0438 (11)	0.0432 (11)	0.0388 (10)	0.0023 (9)	0.0010 (8)	0.0011 (8)
C15	0.0454 (12)	0.0651 (14)	0.0561 (13)	0.0079 (11)	−0.0018 (10)	−0.0045 (11)
C16	0.0441 (12)	0.0653 (15)	0.0565 (13)	−0.0061 (11)	−0.0040 (10)	−0.0041 (11)
C17	0.0480 (12)	0.0471 (11)	0.0403 (10)	−0.0061 (10)	−0.0040 (9)	−0.0013 (9)
C18	0.0594 (14)	0.0578 (13)	0.0395 (11)	−0.0009 (11)	0.0023 (10)	0.0005 (10)
C19	0.177 (4)	0.074 (2)	0.082 (2)	−0.067 (2)	0.061 (2)	−0.0293 (16)
C20	0.117 (3)	0.117 (3)	0.110 (3)	0.043 (3)	−0.017 (2)	−0.064 (2)
C21	0.0519 (12)	0.0467 (12)	0.0491 (12)	−0.0089 (10)	−0.0068 (10)	−0.0055 (10)
C22	0.134 (3)	0.0685 (17)	0.0741 (18)	0.0392 (19)	−0.0296 (19)	−0.0313 (15)
C23	0.0626 (14)	0.0628 (15)	0.0626 (15)	0.0128 (12)	0.0165 (12)	−0.0202 (12)
C24	0.0611 (14)	0.0546 (13)	0.0672 (14)	−0.0023 (12)	0.0081 (12)	−0.0114 (12)
C25	0.106 (2)	0.0581 (15)	0.0559 (14)	−0.0167 (16)	0.0171 (14)	−0.0035 (12)
C26	0.0596 (14)	0.0410 (11)	0.0598 (13)	0.0061 (11)	0.0006 (12)	0.0053 (10)

### Geometric parameters (Å, °)

**Table:**

O1—C3	1.245 (3)	C11—H11A	0.9800
O2—C11	1.424 (3)	C12—C13	1.531 (3)
O2—H2A	0.8200	C12—H12A	0.9700
O3—C17	1.431 (3)	C12—H12B	0.9700
O3—H3A	0.8200	C13—C18	1.535 (3)
O4—C21	1.199 (3)	C13—C14	1.538 (3)
O5—C23	1.316 (3)	C13—C17	1.561 (3)
O5—C22	1.436 (3)	C14—C15	1.527 (3)
O6—C23	1.175 (3)	C14—H14A	0.9800
O7—C26	1.208 (3)	C15—C16	1.529 (4)
O8—C26	1.320 (3)	C15—H15A	0.9700
O8—H8B	0.8200	C15—H15B	0.9700
C1—C2	1.329 (5)	C16—C17	1.543 (3)
C1—C10	1.500 (5)	C16—H16A	0.9700
C1—H1A	0.9300	C16—H16B	0.9700
C2—C3	1.433 (5)	C17—C21	1.532 (3)
C2—H2B	0.9300	C18—H18A	0.9600
C3—C4	1.423 (4)	C18—H18B	0.9600
C4—C5	1.344 (3)	C18—H18C	0.9600
C4—H4A	0.9300	C19—H19A	0.9600
C5—C6	1.504 (4)	C19—H19B	0.9600
C5—C10	1.511 (3)	C19—H19C	0.9600
C6—C7	1.524 (4)	C20—H20A	0.9600
C6—C20	1.529 (4)	C20—H20B	0.9600
C6—H6A	0.9800	C20—H20C	0.9600
C7—C8	1.525 (3)	C21—C22	1.486 (4)
C7—H7A	0.9700	C22—H22A	0.9700
C7—H7B	0.9700	C22—H22B	0.9700
C8—C14	1.522 (3)	C23—C24	1.490 (4)
C8—C9	1.540 (3)	C24—C25	1.476 (4)
C8—H8A	0.9800	C24—H24A	0.9700
C9—C11	1.544 (3)	C24—H24B	0.9700
C9—C10	1.576 (3)	C25—C26	1.478 (3)
C9—H9A	0.9800	C25—H25A	0.9700
C10—C19	1.541 (4)	C25—H25B	0.9700
C11—C12	1.528 (3)		
			
C11—O2—H2A	109.5	C8—C14—C13	113.46 (17)
C17—O3—H3A	109.5	C15—C14—C13	103.62 (16)
C23—O5—C22	116.7 (3)	C8—C14—H14A	106.1
C26—O8—H8B	109.5	C15—C14—H14A	106.1
C2—C1—C10	124.5 (3)	C13—C14—H14A	106.1
C2—C1—H1A	117.7	C14—C15—C16	104.76 (18)
C10—C1—H1A	117.7	C14—C15—H15A	110.8
C1—C2—C3	121.0 (3)	C16—C15—H15A	110.8
C1—C2—H2B	119.5	C14—C15—H15B	110.8
C3—C2—H2B	119.5	C16—C15—H15B	110.8
O1—C3—C4	121.3 (3)	H15A—C15—H15B	108.9
O1—C3—C2	121.3 (3)	C15—C16—C17	106.55 (17)
C4—C3—C2	117.4 (2)	C15—C16—H16A	110.4
C5—C4—C3	124.0 (3)	C17—C16—H16A	110.4
C5—C4—H4A	118.0	C15—C16—H16B	110.4
C3—C4—H4A	118.0	C17—C16—H16B	110.4
C4—C5—C6	123.5 (2)	H16A—C16—H16B	108.6
C4—C5—C10	120.9 (3)	O3—C17—C21	108.18 (17)
C6—C5—C10	115.6 (2)	O3—C17—C16	111.29 (18)
C5—C6—C7	107.9 (2)	C21—C17—C16	113.85 (17)
C5—C6—C20	115.6 (2)	O3—C17—C13	107.50 (15)
C7—C6—C20	111.0 (3)	C21—C17—C13	113.13 (17)
C5—C6—H6A	107.3	C16—C17—C13	102.70 (17)
C7—C6—H6A	107.3	C13—C18—H18A	109.5
C20—C6—H6A	107.3	C13—C18—H18B	109.5
C6—C7—C8	114.3 (2)	H18A—C18—H18B	109.5
C6—C7—H7A	108.7	C13—C18—H18C	109.5
C8—C7—H7A	108.7	H18A—C18—H18C	109.5
C6—C7—H7B	108.7	H18B—C18—H18C	109.5
C8—C7—H7B	108.7	C10—C19—H19A	109.5
H7A—C7—H7B	107.6	C10—C19—H19B	109.5
C14—C8—C7	110.58 (19)	H19A—C19—H19B	109.5
C14—C8—C9	107.34 (17)	C10—C19—H19C	109.5
C7—C8—C9	111.67 (17)	H19A—C19—H19C	109.5
C14—C8—H8A	109.1	H19B—C19—H19C	109.5
C7—C8—H8A	109.1	C6—C20—H20A	109.5
C9—C8—H8A	109.1	C6—C20—H20B	109.5
C8—C9—C11	112.95 (16)	H20A—C20—H20B	109.5
C8—C9—C10	112.9 (2)	C6—C20—H20C	109.5
C11—C9—C10	115.20 (18)	H20A—C20—H20C	109.5
C8—C9—H9A	104.8	H20B—C20—H20C	109.5
C11—C9—H9A	104.8	O4—C21—C22	120.7 (2)
C10—C9—H9A	104.8	O4—C21—C17	123.3 (2)
C1—C10—C5	112.1 (2)	C22—C21—C17	116.05 (19)
C1—C10—C19	107.9 (3)	O5—C22—C21	111.4 (2)
C5—C10—C19	108.0 (2)	O5—C22—H22A	109.3
C1—C10—C9	107.9 (2)	C21—C22—H22A	109.3
C5—C10—C9	107.33 (19)	O5—C22—H22B	109.3
C19—C10—C9	113.6 (2)	C21—C22—H22B	109.3
O2—C11—C12	112.71 (17)	H22A—C22—H22B	108.0
O2—C11—C9	109.50 (17)	O6—C23—O5	121.7 (3)
C12—C11—C9	111.83 (17)	O6—C23—C24	125.4 (3)
O2—C11—H11A	107.5	O5—C23—C24	112.8 (2)
C12—C11—H11A	107.5	C25—C24—C23	112.3 (2)
C9—C11—H11A	107.5	C25—C24—H24A	109.1
C11—C12—C13	113.76 (18)	C23—C24—H24A	109.1
C11—C12—H12A	108.8	C25—C24—H24B	109.1
C13—C12—H12A	108.8	C23—C24—H24B	109.1
C11—C12—H12B	108.8	H24A—C24—H24B	107.9
C13—C12—H12B	108.8	C24—C25—C26	115.9 (2)
H12A—C12—H12B	107.7	C24—C25—H25A	108.3
C12—C13—C18	111.91 (17)	C26—C25—H25A	108.3
C12—C13—C14	108.65 (16)	C24—C25—H25B	108.3
C18—C13—C14	111.88 (17)	C26—C25—H25B	108.3
C12—C13—C17	116.09 (17)	H25A—C25—H25B	107.4
C18—C13—C17	108.49 (16)	O7—C26—O8	122.9 (2)
C14—C13—C17	99.21 (16)	O7—C26—C25	124.6 (2)
C8—C14—C15	120.54 (19)	O8—C26—C25	112.4 (2)
			
C10—C1—C2—C3	0.5 (6)	C11—C12—C13—C17	163.05 (17)
C1—C2—C3—O1	178.4 (3)	C7—C8—C14—C15	−53.9 (3)
C1—C2—C3—C4	0.6 (5)	C9—C8—C14—C15	−175.90 (17)
O1—C3—C4—C5	−179.3 (3)	C7—C8—C14—C13	−177.51 (17)
C2—C3—C4—C5	−1.5 (4)	C9—C8—C14—C13	60.5 (2)
C3—C4—C5—C6	−175.7 (2)	C12—C13—C14—C8	−59.0 (2)
C3—C4—C5—C10	1.3 (4)	C18—C13—C14—C8	65.1 (2)
C4—C5—C6—C7	117.5 (3)	C17—C13—C14—C8	179.37 (16)
C10—C5—C6—C7	−59.6 (3)	C12—C13—C14—C15	168.57 (18)
C4—C5—C6—C20	−7.4 (4)	C18—C13—C14—C15	−67.4 (2)
C10—C5—C6—C20	175.5 (3)	C17—C13—C14—C15	46.91 (19)
C5—C6—C7—C8	53.9 (3)	C8—C14—C15—C16	−160.51 (19)
C20—C6—C7—C8	−178.5 (3)	C13—C14—C15—C16	−32.3 (2)
C6—C7—C8—C14	−170.77 (19)	C14—C15—C16—C17	4.3 (2)
C6—C7—C8—C9	−51.3 (3)	C15—C16—C17—O3	−90.0 (2)
C14—C8—C9—C11	−55.8 (2)	C15—C16—C17—C21	147.40 (19)
C7—C8—C9—C11	−177.16 (18)	C15—C16—C17—C13	24.7 (2)
C14—C8—C9—C10	171.28 (17)	C12—C13—C17—O3	−42.1 (2)
C7—C8—C9—C10	49.9 (2)	C18—C13—C17—O3	−169.09 (17)
C2—C1—C10—C5	−0.7 (5)	C14—C13—C17—O3	74.02 (19)
C2—C1—C10—C19	−119.6 (4)	C12—C13—C17—C21	77.3 (2)
C2—C1—C10—C9	117.3 (4)	C18—C13—C17—C21	−49.7 (2)
C4—C5—C10—C1	−0.2 (4)	C14—C13—C17—C21	−166.61 (17)
C6—C5—C10—C1	177.0 (2)	C12—C13—C17—C16	−159.55 (17)
C4—C5—C10—C19	118.6 (3)	C18—C13—C17—C16	73.4 (2)
C6—C5—C10—C19	−64.2 (3)	C14—C13—C17—C16	−43.45 (18)
C4—C5—C10—C9	−118.5 (2)	O3—C17—C21—O4	−147.7 (2)
C6—C5—C10—C9	58.7 (3)	C16—C17—C21—O4	−23.4 (3)
C8—C9—C10—C1	−173.07 (19)	C13—C17—C21—O4	93.4 (3)
C11—C9—C10—C1	55.1 (3)	O3—C17—C21—C22	33.7 (3)
C8—C9—C10—C5	−52.0 (2)	C16—C17—C21—C22	157.9 (2)
C11—C9—C10—C5	176.16 (19)	C13—C17—C21—C22	−85.3 (3)
C8—C9—C10—C19	67.3 (3)	C23—O5—C22—C21	104.9 (3)
C11—C9—C10—C19	−64.5 (3)	O4—C21—C22—O5	−0.8 (4)
C8—C9—C11—O2	−73.8 (2)	C17—C21—C22—O5	177.9 (2)
C10—C9—C11—O2	58.0 (2)	C22—O5—C23—O6	7.7 (5)
C8—C9—C11—C12	51.9 (2)	C22—O5—C23—C24	−175.6 (2)
C10—C9—C11—C12	−176.30 (18)	O6—C23—C24—C25	−13.2 (5)
O2—C11—C12—C13	73.8 (2)	O5—C23—C24—C25	170.2 (3)
C9—C11—C12—C13	−50.1 (2)	C23—C24—C25—C26	173.7 (2)
C11—C12—C13—C18	−71.7 (2)	C24—C25—C26—O7	−24.4 (4)
C11—C12—C13—C14	52.4 (2)	C24—C25—C26—O8	156.8 (2)

### Hydrogen-bond geometry (Å, °)

**Table:**

*D*—H···*A*	*D*—H	H···*A*	*D*···*A*	*D*—H···*A*
O2—H2A···O4^i^	0.82	2.30	3.115 (2)	172
O3—H3A···O7^ii^	0.82	2.13	2.943 (2)	173
O8—H8B···O1^iii^	0.82	1.82	2.640 (3)	176

Symmetry codes: (i) *x*−1/2, −*y*−1/2, −*z*; (ii) *x*+1/2, −*y*+1/2, −*z*; (iii) −*x*−3/2, −*y*, *z*+1/2.

## References

[bb1] Allen, F. H., Kennard, O., Watson, D. G., Brammer, L., Orpen, A. G. & Taylor, R. (1987). *J. Chem. Soc. Perkin Trans. 2*, pp. S1–19.

[bb2] Anderson, B. D., Conradi, R. A. & Lambert, W. J. (1984). *J. Pharm. Sci* **73**, 604–611. 10.1002/jps.2600730507 6376766

[bb3] Bruker (2005). *SMART* and *SAINT* Bruker AXS Inc., Madison, Wisconsin, USA.

[bb4] Flack, H. D. (1983). *Acta Cryst.* A**39**, 876–881.

[bb5] Sheldrick, G. M. (2008). *Acta Cryst.* A**64**, 112–122. 10.1107/S0108767307043930 18156677

